# 

**Published:** 1881-09-20

**Authors:** 


### TABLE OF CONTENTS.

ORIGINAL AND SELECTED ARTICLES.

Conversations upon the Physical and Mental Hygiene of Girlhood, by T. S. Powell, M.D. Atlanta, 328. Remarks on the Germ Theory of Disease, by C H Wagner, M.D., Miss., 332. A Mechanical Larynx, 333. Central Scotoma, 334. Inutility and Dangers of Medical treatment of Epithelioma of the tongue, 336.

ABSTRACTS AND GLEANINGS.

Case of Croup treated by Passing Catheters into the Trachea by the mouth, 337. What is Pyæmia, 338. Can we make a Positive Diagnosis of Pregnancy Previous to the Occurrence of the Audible Sounds of the Fœtal Heart and the Detection of the Fœtal Movements, 340. Hypodermic Injection of Quinia, 341. The Therapeutical Indications of Bromide of Ammonia, 343. Eucalyptus; On the Treatment of onorrhœa, 345. Euonymus Atropurpureus (Wahoo), 346. Sanguis Bovinus Exsiccatus; Trichlorophenol, a New Antiferment, 347. Noma, or Gangrene of the mouth; Viburnum Prunifolium in Threatened Abortion, 348. The Use of the Lancet; An Anæsthetic Car, 349 Opium Habit; Phenol; Duboisia and Atropia; Menthol, 350. Relation of Fœtal to Maternal Circulation; Scletotic Acid; Gastric Absorption; Successful Transplantation of Human Bone, 351. Caroba, Iodoform & Boracic Acid in Syphilis; Glycerine in Gastric Flatulance, Acidity and Pyrosis; Tartrate of Morphia; Eugenol; Thymol, 352.

PRACTICAL NOTES AND FORMULÆ.

Listerine; Metastatic Prostatitis; Antiseptic Treatment of Enteric Fever, 353. Rhus Aromatica in Gonorrhœa; Treatment of Anorexia; Night Sweats; Ulceration of the Os, 354.

EDITORIALS AND MISCELLANEOUS.

Changes and Resignations; News and Miscellany, 355. Prize Essays; The Atlanta Medical and Surgical Journal; Petrolina; Medical Boards of Georgia Abolished, 356. A Bill to Regulate the Practice of Medicine in Georgia, 357. Autopsy in the President's Case, 358. The Conversations of the Senior Editor, etc., 359. Receipts and Special Notices, 360.

